# The Analysis of Marketing Factors Influencing Consumers’ Preferences and Acceptance of Organic Food Products—Recommendations for the Optimization of the Offer in a Developing Market

**DOI:** 10.3390/foods9030259

**Published:** 2020-02-29

**Authors:** Boban Melovic, Dragana Cirovic, Branislav Dudic, Tamara Backovic Vulic, Michal Gregus

**Affiliations:** 1 Faculty of Economics, University of Montenegro, 81000 Podgorica, Montenegro; bobanm@ucg.ac.me (B.M.); dcirovic@ucg.ac.me (D.C.); tassabacc@ucg.ac.me (T.B.V.); 2 Faculty of Management, Comenius University in Bratislava, 82005 Bratislava, Slovakia; michal.gregusml@fm.uniba.sk; 3 Faculty of Economics and Engineering Management, University Business Academy, 21000 Novi Sad, Serbia

**Keywords:** organic food, market, consumer preferences, product acceptance, sensory properties, optimization

## Abstract

Considering the benefits of the organic production system, it is recognized as one of the main drivers of future economic development. However, the imbalance between demand and supply at the local market level represents one of the serious obstacles that prevents its future growth. Therefore, this article examines the key factors related to the main elements of the offer that have the strongest impact on consumer preferences and acceptance of organic food products. In that sense, organic product, price, distribution channel, and promotion are considered the main elements of the offer and are analyzed in this paper from the consumer preferences perspective. Further, this article provides insight into some of the sensory properties of the offer that are important to consumers. Finally, it gives recommendations for optimization of the offer on the organic food market based on the analysis of the influence of each of those elements (product, price, distribution, and promotion) on consumer acceptance of organic products and making purchasing decisions. The data were collected using a questionnaire, and analyzed using the structural equation model (SEM). The results revealed that price and promotion have the strongest impact on consumer acceptance and buying decisions. Further analysis revealed that attitudes towards organic food products, price/quality ratio, distribution barriers, and modern media as a promotion instrument are the factors that have the most significant impact on consumer perception and attitudes towards the available market offer. These findings can help producers and other decision makers to better understand what creates added value of the organic food products in consumers’ mind and therefore make an offer that is in line with their expectations and preferences, which is recognized as one of the main prerequisites for the acceptance and purchase of organic food products.

## 1. Introduction

Organic production, as a specific system that sustains the health of soil, ecosystems, and people [1,2], appeared as a result of different types of concerns, amongst which the excessive pollution of the environment, imbalance of ecosystems, and human health protection are most important [3,4]. Compared to conventional production, it is an innovative production system that maximizes the performance of renewable energy sources; remarkably reduces the emission of CO_2_, soils, metals, and other harmful substances into the environment; and optimizes nutrient and energy flow in agroecosystems [2]. Additionally, organic production implies the prohibition of the use of pesticides, genetically modified organisms, food additives, and other potentially harmful substances, which results in the production of healthy products of high nutritional value [1,2]. Therefore, fostering organic production has a significant contribution to solving some of the main problems of the global modern society, including obesity (especially amongst young people), environmental pollution, unsustainable production, and the necessity of giving assistance to local farm producers [1,2,3,4,5]. The organic production development has been fostered by income growth and a growing awareness of people about the importance of healthy lifestyles. It is based on several principles, whose implementation ensures the production of safe and healthy products while fully preserving the environment, its natural resources, and fostering equity, respect, and justice throughout society [2,3,4]. Therefore, it is recognized as one of the main drivers of future agricultural and overall economic development on sustainable grounds in many developing and developed countries. 

Although the estimated value of the organic food market reached 97 billion US dollars in 2017, it is characterized by significant inequalities in growth rates, and reached a level of development in different countries [6]. About 90% of all organic products are sold in North America and Europe. Observed by countries, the biggest market of these products is in the USA, followed by Germany, France, and China. On the other hand, the vast majority of organic producers are located in less developed countries in Asia, Africa, and Latin America [6]. An additional obstacle to the intensive development of this sector is reflected in the fact that a small number of loyal consumers buy most of the total organic products produced, while other consumers buy them less frequently and in small quantities. That is why this market still retains some of the niche market features. These data clearly indicate that there is a serious imbalance between supply and demand in the individual geographic markets, caused by a misunderstanding of consumer preferences and key factors that influence the acceptance of organic food products in the market, which is a very important obstacle to the further development of organic food production. Therefore, harmonization of the supply with consumer preferences at the level of local markets is imposed as a necessary prerequisite for its further development. In order to achieve that, it is necessary to optimize the elements of the offer based on the results of more detailed surveys on consumer preferences, as well as on factors that influence the acceptance of organic food products and the consumer decision to purchase them. 

However, in order to adjust the offer to the characteristics of demand in the developing organic food market, it is necessary for producers to achieve specific knowledge of marketing policies and marketing mix. Marketing mix optimization, among other things, includes the adjustment of product features, price level, distribution channel strategy, and promotion to the consumer preferences. In that sense, optimization of the offer that will foster demand on the developing organic market can be achieved through optimization of the marketing mix. 

It is common in theory that product, price, distribution channels, and promotion are regarded as the main instruments of the marketing mix in any market, including organic. That is why the optimization of these elements can be regarded as the optimization of the total offer [7,8]. Therefore, key marketing mix elements are the focus of research in this paper. However, previous studies regarding these instruments on the organic food market mostly included the analysis of consumers’ perception of one of them. However, there is a lack of research comparing the relative strength of the influence of each of these elements on consumer acceptance of organic food products and purchasing decisions, especially in developing markets. Additionally, although there are certain studies that investigate how different factors (such as health consciousness, price sensitivity, buying habits, etc.) influence consumer perception of the offer on the organic market [9,10,11,12,13], according to the authors’ knowledge, there is a lack of research analyzing the relative significance of each of those factors. 

Therefore, considering all the factors mentioned above, as well as the importance of fostering local organic markets’ development, the aim of this paper was to fill these two gaps. First, this research identifies some of the factors that have the strongest influence on consumer perception of each of these main elements of marketing on the developing organic food markets in general. Secondly, it analyzes which of the mentioned elements (product, price, distribution, or promotion) has the strongest impact on the acceptance of organic food products (expressed through the consumer’s decision to buy them). Additionally, this study also gives insight into the relative importance of some of the sensory properties of these products and their influence on consumer perception of the offer and the overall acceptance of the organic food products in developing markets. 

The data analyzed in this paper were collected through a survey conducted in Montenegro. The motive for conducting research in this country is based on two reasons. Montenegro was one of the first countries in the world that was declared as an ecological state [14]. This, among other things, shows its potential for sustainable development and sustainable production, such as organic. However, despite its great potential, the organic food market in this country is still in its early stages of development. Therefore, it is necessary to understand what drives consumer behavior on the food market and what the main factors that influence the acceptance of organic food products are, in order to foster the development of this sector. Additionally, the obtained results can be useful for researchers and decision makers in other countries with similar production possibilities and an underdeveloped organic market.

Besides the theoretical contribution, the obtained results can help organic producers to better understand the main factors that shape consumer behavior on the developing organic food market and better allocate limited resources on each of the investigated elements of the offer, therefore creating more successful market strategy at the local and global level. Additionally, the obtained results can also help policy makers to better understand factors that drive organic market growth as well as those that prevent its further development, therefore allowing them to create and implement measures that will have significantly positive effects. However, it should be noted that fostering organic food production in developing markets can have significantly positive consequences that go beyond this economic sector. First of all, organic production is a system based on natural renewable resources that has no negative impact on the environment [1,2]. Therefore, it encourages economic development on sustainable grounds and a reduction of environmental pollution as one of the biggest global problems. Secondly, fostering organic agriculture development at the local level can help to reduce the problem of poverty and food waste, as it would increase employment rates and the production of food in the places of its consumption [2,15]. Finally, there are recent studies indicating that frequent consumption of organic food reduces the risk of obesity, which is also an important health issue that the global population faces nowadays [16]. 

## 2. Literature Review 

### 2.1. Theoretical Background

Research conducted by Kriwy et al. as well as the research of Kashani-Nazari et al. showed that women are more prone to purchasing organic products compared to men [17,18]. On the other hand, the study of Kokmaz et. al. obtained the opposite results, indicating that men are more prone to purchase these products compared to women [19]. Although most of the studies showed that consumers who are younger, highly educated, and have higher level of income are more likely to buy organic products [17,20,21,22,23], there are some studies that revealed different results, showing that age, profession, and education do not have a significant impact on consumers’ acceptance of organic food [19,24]. Considering the fact that that demographic analysis did not provide the information needed for successful targeting of organic food consumers, many authors applied segmentation analysis in order to identify the characteristics of different segments of organic food consumers. The results revealed that the same segments of organic food consumers cannot be found in all investigated markets, i.e., that organic food consumers have different demographic, psychographic, and behavioral characteristics, observed in different markets and different countries [9,20,25,26]. Therefore, different studies were conducted regarding consumer perception and barriers that prevent them from buying these products in order to better understand what drives the consumption of organic products. Currently, there is a growing research body on the motives and barriers that determine the behavior of organic consumers and influence their decision-making process. Most of those studies showed that consumers are faced with quite similar motives and barriers when purchasing organic food products, although the degree of their influence can significantly differ. The most important reasons for buying organic food products can be divided into three groups [27]: Avoiding the negative properties of conventional food products (such as the use of different harmful chemicals in production), unbiased features of organic food (such as safety), and positive features that consumers believe these products have (better taste, bigger nutritional value compared to conventional products, etc.). This is confirmed by most of the studies conducted in different markets that suggest that the most important motives for buying organic food are related to consumer perception of these products as healthy, tasty, safe, and of high nutritional value [9,10,20]. Additionally, consumers believe that organic production ensures protection of the environment, animal welfare, and preservation of natural resources [9,10,11,13,20,28]. Additionally, it should be pointed out that health and environmental concerns are far more important than other reasons, as well as the fact that the relative importance of each of these reasons did not significantly change over time [29]. A study conducted by Kereklas [30] showed that, unlike the purchase of conventional food products that is mostly based on personal considerations and habits, the acceptance of organic food products is under the significant influence of altruistic concerns, as well as under the influence of social pressure and social approval. However, the impact of these factors is rather indirect and is of less importance for price-conscious consumers [31,32]. Although most consumers have a positive attitude towards organic food products, there is only a small number of them who act in accordance with those attitudes, i.e., there is a far lower number of consumers who often buy these products. Therefore, the link between attitudes and buying intentions is much stronger than the link between intentions and the real behavior of consumers [33]. The reasons for such a condition can be found in the barriers that consumers face when buying organic food. Most authors state premium prices as the most important barrier, followed by the narrow range of products, poor availability in stores, consumer mistrust in certification labels, lack of time in the buying process, as well as negative attitudes towards organic products caused by a lack of information and satisfaction with conventional food [24,33,34,35]. 

### 2.2. Key Marketing Determinants Affecting Organic Food Consumption

A number of authors conducted research related to consumer perception of one specific marketing instrument as an important element of the offer (product, price, distribution, or promotion), in order to make suggestions for improvement of the offer on the organic food market [36,37,38,39,40,41,42]. Thanks to the specificities of organic production, organic food products have additional value that motivates consumers to buy them. However, that additional value cannot be experientially confirmed, which is why consumers search for ways in which organic food products can be distinguished from conventional ones [36,37,38]. This points out the importance of the visual cues of the product that can positively or negatively affect consumers’ senses and perceptions of the product features. However, although certification is very important for consumer confidence, it is still uncertain which visual attributes organic food products should have and what is the role of packaging. The study of Enax revealed that it is of great importance to consumers, who find products with recognizable packaging healthier and tasty [39]. Similar results were obtained by Puelles et al. [40], emphasizing the difference between producers’ and distributors’ brands. The producers’ brands are perceived as of better quality compared to distributors’ brands, while distributors’ marks are directed at the group of price-conscious consumers. A study of Aschemann-Witzel investigated the importance of nutrition and health claims visible on packaging and their effects on consumers’ perception of the sensory properties of organic food products [41]. The results revealed that products with claims were neither significantly more preferred nor rejected when dealing with regular or loyal organic food consumers. However, highlighting these claims on packaging had significantly positive effects on occasional buyers. On the other hand, research conducted by van Herper et al. [42] obtained the opposite results. It revealed that packaging of any type can have negative effects on organic food sales (especially on organic fruits and vegetables), pointing out the fact that offering products without packaging increases the choice of consumers and that this effect is not limited to organic products only. Considering the results of previous studies, it still remains unclear what the visible features of organic food products that (besides of formed attitudes) have the strongest effect on consumer perception and senses are, and how much those effects are important for the acceptance of organic food products. 

Price is considered to be one of the main obstacles that consumers face in the process of making buying decisions in the organic food market [24,33,34,35]. Bearing in mind that these products have premium prices due to higher production costs and limited supply [43], as well as the fact that its added value is intangible, Iyer et al. [31] suggest that the emphasis should be placed on the promotion of current and future benefits, especially when targeting price- and value-conscious consumers. Similarly, Bezawada et al. [44] point out that a reduction of regular prices and increasing assortments are very effective for non-core organic food consumers, and they also stimulate purchasing by core consumers. This is especially evident in the sales of organic food products with a high purchase frequency, as well as for those products that come directly from farms. However, promotional price cuts do not have significant effects on the purchase frequency of this type of product [44,45]. Marian et al. obtained similar results [46], showing that the least purchasing frequency is achieved for the most expensive organic brands. This implies that when consumers have some other quality cue (such as an organic certification label), they perceive higher prices only as a cost, not as an indicator of additional product value. Previous findings suggest that price reduction would be very effective for the stimulation of organic food purchasing. However, it still remains unclear whether consumers perceive the premium prices of these products as unjustified, or if these prices are a purchasing barrier due to insufficient income for most consumers. Answering this question is one of the main prerequisites for creating a successful business strategy on the organic food market. 

An additional important barrier to organic food market growth is insufficient development of distribution channels [33,34,35]. However, different channel types attract different segments of consumers. Specialized organic stores are especially important for attracting core organic consumers [47,48], as these stores have a wide range of certified organic food products. On the other hand, although the internet can be an efficient channel for organic sales aimed at young people [48,49], the development of conventional stores is especially important for attracting non-core organic consumers, even though there is also a small percentage of loyal organic buyers who purchase in this type of store too [43,45]. Although consumers form an attitude towards groceries and supermarkets not only according to their personal habits but also according to the range of products that these shops sell [50,51], including organic products in the offer can have a positive influence on retail image, through improving its social dimension. Additionally, it can have positive effects even on the image of low-pricing outlets. According to the study of Diallo et al. [52], although organic products have premium prices, customers do not see a link between organic products and the store price level, probably because they believe that organic premium prices are related to production costs and high quality, and not related to the price strategy of the store. The results of previous studies suggest that consumers perceive an insufficient development of distributive channels as one of the main barriers to further development of the organic food market. However, the obtained results do not indicate whether consumers perceive the lack of these products in their favorite grocery stores as the main problem, or the consumers’ inability to find organic food products at any other type of store despite the effort they are ready to make when purchasing these products is an even more important barrier. 

Previous studies related to the promotion of organic food products were focused on creating messages line with the motivations of organic consumers. Kereklas at al. [30] revealed that the promotional messages featuring both altruistic and egoistic appeals are most effective when targeting organic consumers. Similar results were obtained by several other authors [53,54]. However, although some studies suggest that social networks and other Internet-based channels could be efficient instruments for the promotion of organic food products [49,55], it is still unclear whether consumers prefer traditional or modern media as a source of information about this type of product and what the relative impact of different promotion channels on consumer preferences and acceptance of this product type is. Considering all factors previously mentioned, this paper fills one of the literal gaps regarding marketing mix instruments that are addressed above, as basic elements of the offer on the developing organic food market.

### 2.3. Conceptual Framework

The results of previous studies revealed that the existing imbalance between demand and supply at the local market level represents a very important obstacle to the further development of the organic food market in general [6]. Additionally, consumers state that there are significant deficiencies regarding all main elements of the offer (addressed in this paper) that prevent them from buying organic food products, despite the positive attitudes they have towards them [23,32,33,34]. The authors consulted previous research in the field in order to consider all relevant factors that can influence consumers’ perception towards four main marketing elements and their purchasing decision, in order to formulate the research questions and the conceptual model (some of the most important are presented in Table A1 given in Appendix A of this paper). The previous literature review indicated that there are many factors influencing consumer attitudes and perception of these elements, but which of them has the prevalent role in that process has not been investigated enough. Therefore, the first research question in this paper is formulated as follows:

**RQ1:** What are the factors that have the strongest influence on consumer perception of each segment of the offer in the developing organic food market?

Additionally, although previous studies investigated consumer perception towards some of these elements, which of them has the strongest impact on consumers’ buying decision has not been investigated enough. Therefore, the second research question is formulated as follows:

**RQ2:** Which element of the offer, viewed from the marketing aspect (product, price, distribution channel, or promotion) has the strongest impact on consumer acceptance of organic food products, expressed through the decision to buy them?

**Figure 1:**
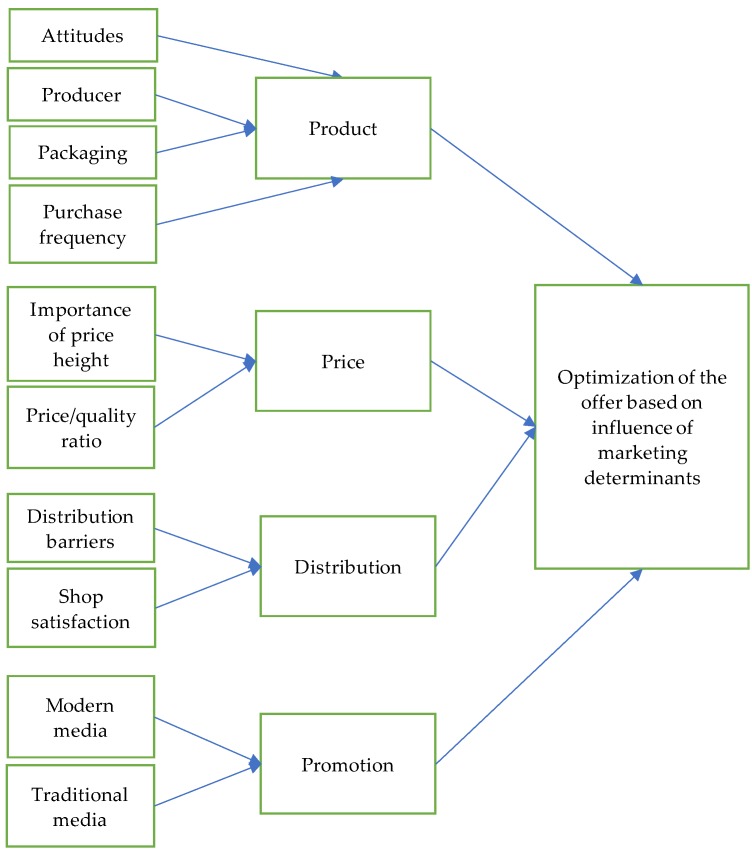
Conceptual model of research.

The conceptual model was formed based on the research questions and it is given in Figure 1 presented below.

Our basic structural model contains three main constructs: (1) Factors that influence consumer perception of the stated elements of the offer (product, price, distribution channels, and promotion), (2) the consumer perception of these elements, and (3) the acceptance of products expressed through purchasing decisions. 

Attitudes towards organic food products, producer, packaging, and frequency of purchase were identified as factors that influence consumers’ senses and perception of the organic food products as the first element of the offer. The importance of price and the price/quality ratio were identified as factors that influence consumer perceptions of price. The distribution barriers (understood as the inability of consumers to find organic products in retail outlets in general) and consumer satisfaction with shops were identified as factors that influence consumer perceptions of distribution as an element of the offer. Finally, the modern media and the traditional media were identified as factors that influence consumer perceptions of promotion and its efficiency in fostering an acceptance of organic food products. In that sense, Internet-based media, such as social networks, email advertising, mobile marketing, pop-up advertising, etc., are considered as modern media, while TV, radio, newspapers, magazines, flyers, etc. are considered as traditional media [56,57]. While modern media are used for direct marketing communication and more precise targeting at relatively low prices, traditional media are still the best way of spreading information and gaining a large number of potential consumers, but the relative impact of these types of media varies depending on the industries of advertisers [58,59]. 

Each of these factors was chosen based on the previous research in the field related to their impact on the consumer perception of the offer on the food market. Therefore, each of them has a certain (positive or negative) influence on consumer perceptions and attitudes towards the named elements of the offer. On the other hand, consumer perceptions and attitudes towards these elements affect their acceptance of organic food products, i.e., purchasing decision. The causality between the identified factors and consumer perceptions and attitudes towards the four main elements of the offer, as well as the causality between consumer perceptions and attitudes towards these elements and the acceptance of organic food products expressed through purchasing decision, is what the authors explore in this paper. A better understanding of the causality and strength of the link that exists between these constructs will give insight into the actions that should be taken towards optimization of the offer, in order to foster the purchasing of organic food products and their acceptance. 

## 3. Materials and Methods

Bearing in mind the research goals, formulated research questions, methods, and results of previous studies in this field, as well as their previous research experience in the organic food and marketing, the authors developed a questionnaire (which is given in Appendix B of this paper). A pilot survey was realized to examine the validity of the content of the questionnaire and was conducted on 40 respondents. Based on their suggestions, the final form of the questionnaire was prepared. It consisted of 22 questions and it was prepared in the Montenegrin and English language. The questionnaire included two types of questions: The multiple-choice question and a 5-point Likert scale. The questionnaire was formed considering the aims of research and the formulated research questions, in order to provide the data needed for measurement of the influence of factors addressed in the model. Most of the questions from the final questionnaire (questions 6, 7, 8, 9, 10, 11, 12, 20, 21, and 22) were related to the factors that influence consumer perceptions of organic products as a key element of the offer: The attitudes towards organic food products, the producer, the product packaging, and the purchase frequency. Considering the fact that price is one of the most important purchasing barriers, the questionnaire also contained questions related to factors affecting consumer perceptions of price: Price/quality ratio and the importance of price (questions 10, 11, 12, and 16). As the development of distribution channels is one of main prerequisites for organic food purchasing, questions 10, 12, 13, 14, 15, and 20 were related to factors affecting consumer perceptions of these channels (distribution barriers and shop satisfaction), while questions 10, 17, 18, and 19 were related to modern and traditional media in order to determine which of them has a stronger impact on the consumer perceptions of promotion. 

In cooperation with the chamber of commerce in the mentioned country, the questionnaire was forwarded online through the bases of corporative group mails of retail companies to the 3530 potential respondents. The poll lasted for 30 days and it was conducted in the third quarter of 2019 in Montenegro. In total, 1634 respondents took part in the survey, which represents an answer rate of 46.3%. However, the total sample consisted of 1051 respondents. It is formed of respondents who buy organic food products at least a few times a year and their selection was made by using a filter question in the first part of the questionnaire, regarding the frequency of organic food product purchasing. So, if the questionnaire was completed by the respondent who never buys organic products, it is not considered valid. In that sense, all 1051 accepted questionnaires are valid and represent a validity rate of 64.3%. Based on the explanation of the sampling procedure, we can conclude that this is a stratified random sample. The motive for this selection is found in several reasons. First of all, it is expected that consumers who purchase organic food products at least sometimes are better informed about the principles of organic production and the characteristics of organic food products. Secondly, these consumers have more information on the real features of the available offer in the organic food market, and therefore can give more precise answers. Finally, compared to the non-consumers, it is easier to encourage the existing consumers to buy these products more often and in larger quantities. 

Considering the fact that based on the results of previous studies it is not possible to determine the level of influence of education, occupation, income level, or other consumer demographic characteristics on the purchase frequency of organic food products, the stratified random sample method was applied. This choice of sample was also supported by the fact that there are only a few studies in Montenegro related to the organic food consumer, and the obtained results did not create a reasonable base for choosing another type of sample. Therefore, this method allows respondents of different demographic and psychographic characteristics to be represented in the sample. Additionally, considering the fact that there is a lack of research regarding this topic in Montenegro, there are no available data about existing specific consumer segments in the organic market that could be used as a basis for different sampling. Hence, the application of a random sample method provides an acceptable degree of probability that the sample structure corresponds to the structure of the population. Additionally, we point out the fact that national statistics body, Statistical Office of Montenegro, conducts research of a whole population based on a sample of 1000 respondents [60], which also confirms the representativeness of the sample in this study. In terms of gender, 60.3% of the respondents were female while 39.6% of them were male. This sample structure regarding gender is representative, considering the fact that women do the purchasing of the food products more often compared to men. Additionally, this gender structure of the sample is very similar to the gender structure of the population in Montenegro [60], which also confirms its reliability. Organic food products are suitable for all ages, which is why the sample included all respondents who are over 15 years old (younger consumers rarely purchase themselves). A more detailed overview of the respondents’ characteristics regarding their age, level of education, level of income, and purchase frequency is given in Table 1. 

Further data analysis was preceded by determining the reliability of the survey results obtained on the basis of a questionnaire. The reliability coefficient of the whole questionnaire is 0.868 and represents the acceptable value of this coefficient in social science research. The data were analyzed using structural equation modeling (SEM). The validity of this model specification can be tested using a large number of tests. The most commonly cited tests refer to the model validity indexes: Goodness of fit index (GFI) and adjusted goodness of fit index (AGFI). The obtained values of these indexes for the model given in this paper are presented in Table 2 given below. 

In order to consider this model valid, these two indexes should have values equal or greater than 0.9. This condition is fulfilled for the presented model. Table 2 also gives the result of another widely accepted test, the root mean square error of approximation (RMSEA) index, whose recommended maximal value is 0.1. In our model it takes the value of 0.08, which is also acceptable. Thus, the results of the structural equation model (SEM) evaluation can be taken as valid. The obtained results are presented in the following section. 

## 4. Results

**Figure 2:**
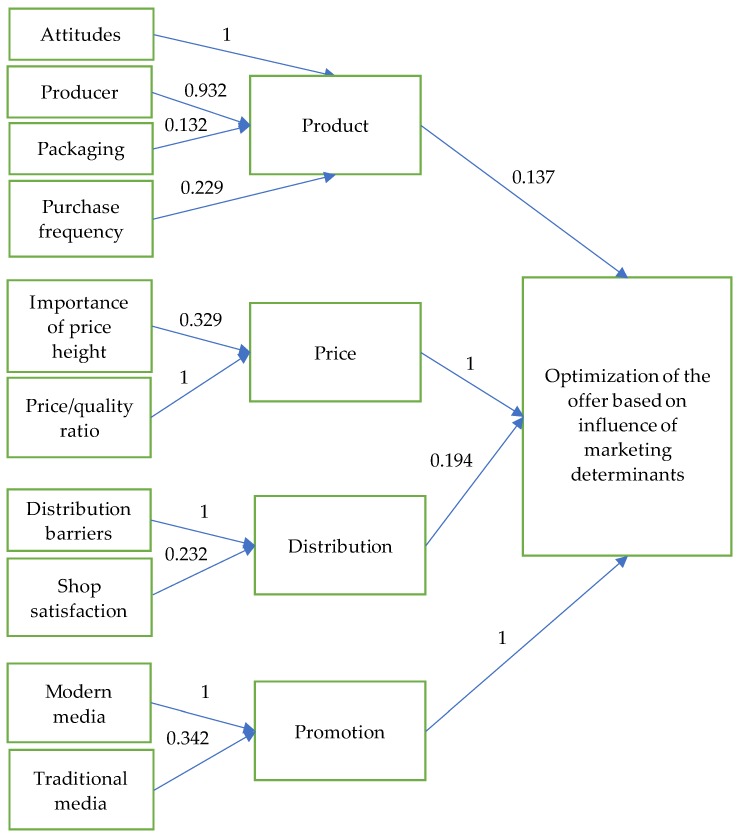
Structural equation modeling results.

The structural equation model (SEM) comprises a set of statistical methods designed to explain the complex relationship between one or more independent variables and one or more dependent variables. In this model, we used the rank-based strategy, proposed by Woods [61], to select anchor items (variable whose path relation will have value 1 as a constraint). Through the process of estimation of the structural equation model in this paper, 10 factors were identified that represent the elements of the offer. The factors were formed on the grounds of the joint influence of 45 independent variables, which were derived from the questionnaire and grouped in four new factors on the basis of their common characteristics. In evaluating the structural equation model, the factor that displays the characteristics of the respondents had to be excluded from the model because the parameters of this factor were not statistically significant. Thus, the structural equation model includes 10 factors. Factors that represent respondents’ views, producer characteristics, packaging of organic food products, and purchase frequency were presented by their common factor named *Product*. Factors that indicate the importance of price height when buying organic food products and the price/quality ratio are presented by a common factor *Price.* The factor *Distribution* represents the impact of barriers in the distribution of organic food products and store satisfaction. The fourth common factor named *Promotion* indicates the impact of promotional activities (using traditional or modern media) when making a decision to buy organic food products. The regression coefficients obtained in the SEM model are presented in Figure 2, which is given below. 

The values of the standardized regression coefficients are given above the straight lines in the path diagram. The higher their value, the more the specific factor is considered a good indicator of the decision to buy organic food products and, therefore, an indicator of good product acceptance. 

In order to justify the results of the SEM analysis, we examined whether the regression coefficients in SEM are statistically significant, as well as the validity of the specification of the defined model. The estimation results of this model are given in Table 3. 

All the regression parameters of the estimated structural equation model are statistically significant. This is confirmed by the corresponding probability values of the regression coefficients, which are marked with asterisks. Thus, with an error risk of 5%, all regression coefficients are statistically significant. For the estimation of SEM for each factor, a limit is defined according to which the value of the regression coefficient between the observed factors is 1. Since the value of a given regression coefficient is predefined, the statistical significance is not tested for it. In our model, value 1 is defined for the coefficients that relate the impact of attitudes and *product*, then the impact of the distribution barriers on the factor *distribution*, the influence of the factor *promotion* on the purchase decision, and the effect of *price* on the purchase decision.

Another advantage of the SEM model is the fact that it can be used to determine which factor has the strongest influence on respondents’ decision to buy organic food products. Based on the estimated values of the regression coefficients for individual factors, it is concluded that the formation of the factor *product* was influenced the most by the variable that reflects customer attitudes towards organic food products. These findings are also supported by the results of the descriptive statistics in this research, which reveal that the main motivation for buying organic food products mostly relies on positive attitudes towards them and to a much lesser extent on the properties of these products that actually affect consumer senses (such as the visual appearance, taste, packaging, label, etc.). About two thirds of the respondents (58%) stated that organic food products are healthier compared to conventional ones. Additionally, 71% of them indicated the positive influence of these products on health as the main motive for their purchase. The additional reasons for purchasing are the nutritional value of the organic food products, environmental protection, animal welfare, and habit. On the other hand, consumers state that the taste of the organic food has the strongest effect on their perception, compared to other features that can be experientially verified. For 11% of them, this is, at the same time, the most important reason for purchasing. Other features of these products that can affect consumer senses (such as visual appearance, labeling, etc.) are of less significance for the purchase decision. 

For the factor *price*, the most significant variable is the one that represents the price/quality ratio of organic food products. They suggest that if consumers perceive organic products as being high quality, they will be ready to pay premium prices for them. This points out the importance of educating and informing consumers about all the benefits of organic production for them as individuals and for the whole society. In that sense, the results of the descriptive statistics should be pointed out, which revealed that only 9.1% of consumers stated that they are not willing to pay premium prices for organic food products. Most of them were ready to pay 0% to 20% higher prices for organic products, compared to conventional (43.4% of consumers), while almost a third of respondents (28%) stated they are willing to pay 20% to 40% higher prices for this type of food product. These results reveal that premium prices are not an insuperable barrier to a wider acceptance of organic food products, and its significance is under the influence of information and the education of consumers towards an organic production system and all the benefits of this type of food.

In the case of the factor *distribution*, the most important was the variable that represents distribution barriers. Additionally, these results show that consumers do not expect that organic food products will always be available in their favorite stores (as the regression coefficient of this factor is smaller: 0.232), which means that they are ready to make an additional effort of going to another store, when purchasing these products. The descriptive statistics of the consumer preferences towards stores where organic products should be available revealed that most of them (57.7%) consider large supermarkets as the establishments where these products should be represented, while 20.4% of them consider that there should be more specialized organic stores in the market. 

The factor *promotion* is mostly defined by a variable, which represents modern methods of promotion, suggesting that the promotion of organic food products in developing markets should be mostly conducted using modern media, such as social networks, Google adwords, banners, emails, and other Internet-based promotion channels. 

The results of the evaluation of the structural equation model, i.e., the values of the estimated coefficients in the model, showed that *price* and *promotion* have a decisive influence on the consumer acceptance of organic food products and on the decision to buy them, while the influence of the factors *product* and *distribution* is less significant in developing organic food markets. These results are not so unexpected considering the purchasing power of the respondents who participated in the survey, the fact that organic products are currently mostly attractive to buyers with a higher income and higher education, as well as the fact that promotional activities disseminate information about the importance, quality, and availability of these products. Therefore, price and promotional activities must play a key role in the process of creating consumer perception of organic food products and fostering acceptance of these products in the society in the developing markets. On the other hand, for the buyers of the organic food products in such markets, the most important thing is the fact that these products meet the criteria of being truly "organic", which is why they pay very little attention to the other characteristics of these products, such as packaging or the manufacturer. Promotional activities in developing markets aim to inform customers where the organic food products are available, which is why the distribution channels also do not play such an important role in the process of making decisions to purchase these products. 

In addition to the previous analysis, the authors also conducted the SEM model in order to test if there are differences in the significance of the investigated factors between men and women. The obtained results of the regression coefficients are given in Table 4. 

The results of the SEM model obtained by using the gender as a control variable indicated that there are similarities but also certain differences in the way in which identified factors affect the purchasing decision of men and women in the organic food market. The results presented in the table explain that male consumers are highly influenced by the frequency of consumption, price/quality ratio, and distribution barrier when making a final decision to buy organic food products. Therefore, it is very important to diminish distributive barriers and explain all the benefits that consumers get for money (especially when it concerns the type of products with the biggest purchase frequency) in order to foster men to purchase these products more often and in larger quantities. Similarly, women are also mostly influenced by the price/quality ratio and distribution barriers, but traditional media has a significant impact on their purchasing decision too while it has no impact on men. Both men and women are price sensitive, which is why price is regarded as a very important obstacle to the organic food purchase. Additionally, they are positively influenced by promotion through modern media. This indicates that the results of the previous general SEM model can stand for both genders, confirming its reliability.

## 5. Discussion

Although consumers make purchasing decisions considering all elements of the offer as a whole, the previous analysis revealed that price and promotion have the strongest influence in that process on developing markets. The results of the structural equation model applied in this research showed that product does not have such a strong influence, compared to price and promotion. These results are somewhat surprising, considering the fact that earlier studies confirmed the specificities of the organic production system and objective features of organic food products (such as their nutritive value, health benefits, environmental benefits, etc.) as the main motives for buying these products [9,10,11,13,20,28,29]. However, the explanation could be found in the analysis of the significance of the factors that influence consumer perception of organic food products. The previous analysis showed that attitude is the factor that has the strongest impact, followed by producer. Such results are expected considering the fact that previous studies have shown that consumers evaluate the value of organic products mostly on the basis of their objective properties, resulting from the specificity of their production method (such as health safety, nutritional value, production process that is not harmful for the environment, etc.), so that the additional features of these products, such as the packaging and visual appearance, are not of great importance [7,8,9,10,12,19,27,28,62,63]. These additional features gain importance mostly when there is no reliable evidence that given food products are organic [38,64]. This reveals that consumers view all types of organic food products as of a similar level of quality, regardless of their packaging and other added features. Therefore, their objective characteristics that affect consumer senses and that can be experientially confirmed are of less significance. However, the most important amongst them is the taste of the product. The information about the producer is also important, as consumers search for cues that certain products are really organic, especially in developing organic markets where other cues are often missing. Therefore, trust in the producer reduces their doubts. However, considering the fact that these products have premium prices and that consumers in less developed markets do not have enough information about these products (regarding all their benefits, the available range, the price range, or the stores where they can be found), positive attitudes towards organic food products do not have such a significant role in the real behavior of consumers.

The fact that price has a strong effect in the process of accepting organic food products is confirmed by previous studies that revealed the price as one of the main barriers [24,33,34,65]. These results are not surprising, considering the fact that these products are more expensive than conventional ones and that they usually have premium prices, while on the other hand, the purchasing power of the consumers in Montenegro is relatively modest. According to the data retrieved from Statistical Office of Montenegro, Monstat, the minimal consumer basket for Montenegro in December 2019 amounted to 646.70 EUR, out of which 271.2 EUR refers to expenditure on food and non-alcoholic beverages, while 375.5 EUR refers to the expenditure on other non-food products and services [60]. On the other hand, the average earnings amount to 520 EUR [60], which implies that consumers are price sensitive first of all due to a low income. However, the analysis of factors that influence consumer perceptions of price revealed that their perception and behavior towards these products strongly depends on the product quality/price ratio and this factor is important for both men and women. In other words, if they consider that the product is of high quality, they will be ready to pay higher prices. Therefore, educating and informing consumers in Montenegro (and in other developing markets) about the organic production concept, as well as about all the benefits of the consumption of organic food products for individuals and for the whole society, is one of the main prerequisites for accepting these products and converting positive attitudes into real purchasing. 

Although previous studies pointed out the significance of distribution channels as one of the main barriers in organic food purchasing [33,34,35,43,45,47,48] and therefore, as one of the most important factors that influence consumer perceptions and purchasing decisions, the data analysis in our study gave the opposite results. Although women are to a certain extent influenced by this factor, it is not of such great importance for consumers in general. This indicates that consumers in developing organic markets do not expect that organic food products should be available in every type of food shop and that those consumers who intend to buy these products are ready to make additional effort in searching for specialized stores where they can find them. However, it is important for them to know exactly where these products can be found. The previously mentioned factor was confirmed by the analysis of the factors that influence the perception of distribution channels. The results revealed that satisfaction with grocery stores does not have a significant impact, while the lack of these products in most of the stores is regarded a very significant factor that prevents their further acceptance and real purchasing in larger quantities. The results of the descriptive statistics showing that organic food products should be available more in the supermarkets and specialized stores are not surprising. Poor distribution channel development is a common problem in most developing organic food markets, such as Serbia or Poland [66,67]. Additionally, supermarkets and specialized stores are the most important distribution channel even in developed and mature organic food markets, such as Germany, France, and Canada, where supermarkets offer large varieties of product types and brands, while the specialized stores are usually better adapted to special consumer segments by offering a wide range of products customized and adapted from bulk or small packing containers [42,58]. Therefore, in the mature markets, producers can focus on in-depth development of their organic brand lines instead of fostering the development of distribution channels and generic promotion of organic food products as it should be the focus in emerging and developing markets [45,67,68]. 

The strong effect of promotion on the purchasing decision of consumers is also understandable. As Song et al. found in their research, the additional value of organic food products that makes them different from conventional ones cannot be experientially confirmed. This is why consumers search for ways in which organic food products can be differentiated from conventional ones [36,37,38]. Therefore, a very important role of promotion is to inform consumers of how they can distinguish them from non-organic products [65]. Additionally, in underdeveloped or developing markets of organic products, such as the market in Montenegro, it is expected that consumers are not well informed about the concept of organic production. Hence, promotion has a very important role of educating and informing consumers about the characteristics of organic products, the certification process, the available offer, and all other features that could be significant for them in the process of accepting these products and making purchasing decisions. The analysis in this paper also revealed that modern instruments of promotion in developing markets have a stronger effect compared to traditional media. More precisely, although traditional media has a significant impact on women, it does not influence the purchasing decisions of men. However, both genders are influenced by modern media, which is why this marketing element should be the focus of producers in developing organic food markets. These results are in line with the research of Pechrová and Jesus Medina-Viruel, [49,55], showing that the Internet and other modern promotion tools could be effective in promoting this type of product. However, it should be noted that most respondents in the sample were younger than 40 and had high school or university education, since younger and more educated people tend to use modern media much more than traditional ones. 

Considering all previously mentioned factors, suggestions for marketing mix optimization in the developing organic food market can be made. Although all four marketing mix instruments as main elements of the offer should be significantly improved, the results in this paper reveal that most of the efforts should be made regarding price and promotion. Although consumers exhibit different levels of price sensitivity, they consider organic food prices related to the quality of products. Therefore, decision makers should point out all the benefits of organic farming and organic food consumption. If consumers understand the value of these products, they will be ready to pay premium prices. The fact that a significant number of respondents in the sample had an average income or income below the average confirms that the previous conclusion holds for price-sensitive consumers as well. However, in order to achieve the mentioned goal, promotion should be improved and conducted using predominantly modern media. Considering the fact that in developing markets, consumers mostly lack information about the organic production concept, promotional activities have a very important role in educating and informing consumers about the quality of organic food products and justification of their premium prices. Additionally, through the education of consumers, promotion can have a significant impact on their attitudes towards organic food products (which is in line with our research that promotion has a stronger impact on purchasing decisions, compared to product). Promotional activities also inform consumers about the available offer in the market, as well as about the stores where organic food products are being sold, which explains why distribution does not have such a strong influence on the purchasing decision of consumers. This way, the impact of the price and distribution as two main obstacles for the acceptance of organic food products in developing markets will be removed, or at least reduced. At the same time, by diminishing the impact of these barriers, the prerequisites for further development of the organic market will be fulfilled. Considering the specificities of organic production, the development of this sector will further have a series of positive consequences. First of all, it will have a significant contribution to better allocation of renewable resources, as organic production is a system completely adjusted to natural conditions [1,2]. Secondly, it enables food production in the places of its consumption, therefore contributing to solving problems of food waste and poverty in rural areas that developing countries mostly face [2,15]. Thirdly, fostering organic agriculture can contribute to the efforts in promoting healthy lifestyles and especially in solving the problem of obesity, which is a common problem of numerous countries [2,16]. Finally, fostering the organic food market will enable economic development without a negative impact on the environment [1,4], which is in line with the efforts of many developing countries to achieve economic development on healthy and sustainable grounds.

## 6. Conclusions and Future Research Recommendations

The aim of this paper was to generate recommendations for optimization of the offer on developing organic food markets, based on the analysis of the impact of four main elements of the offer (product, price, distribution, and promotion) on consumer acceptance of organic food products in developing markets. Additionally, in order to better understand the intensity and nature of those effects, this paper investigated which factors have the biggest impact on consumer perceptions of each of these elements. 

The results revealed that price and promotion have the biggest impact on the acceptance of organic food products and consumer purchasing decisions in developing markets, while the impact of product and distribution channels is relatively modest. These results are not so unexpected, considering the fact that organic food products have premium prices, which makes them hardly affordable compared to conventional products. Additionally, promotion has the role of educating consumers about health, environmental, and other benefits of organic production, as well as the role of informing consumers about all the aspects regarding the available offer of these products in the market. This is especially important in developing markets due to a lack of information about the organic production concept and organic food benefits that most of the consumers face. 

The analysis of factors that impact consumer perceptions of each of the addressed elements of the offer gave the additional explanation for such results. This analysis showed that consumers do not make a difference between different types of organic food products. Therefore, factors, such as packaging or frequency of purchase, do not have an impact on their perception of organic food in general. However, having a reliable cue that given products are really organic is very important for consumers, which is why they look for a recognizable organic producer. This points out the significance of giving reliable visual cues to the consumers that will help them to differentiate organic products from non-organic. The consumer perception of price depends on the perceived quality of products and this is very important for both men and women. If they perceive that the product is of high quality, they will be ready to pay premium prices, which is why additional effort should be made in order to justify the premium prices of organic food products. Although consumers do not expect organic food products to be available in most food shops, the poor development of distributive channels is perceived as a significant barrier, which points out the fact that its future development is very important for ensuring the long-term growth of this sector. On the other hand, promotion is expected to inform and educate consumers about all important features of organic food products and therefore diminish the deficiencies of the other three elements of the offer. Although women are more strongly influenced by traditional media, for the best results, it should be mostly based on modern media due to the fact that both genders are influenced by this media type.

Apart from the theoretical factors, this paper has a significant practical contribution. It reveals the relative impact of the main marketing elements in developing organic food markets on consumer purchasing decisions, therefore helping decision makers to allocate limited resources to each of them. Additionally, it analyzed the factors that influence consumer perceptions of each of those instruments, therefore helping the decision makers to better understand what creates the added value of the offer in the consumers’ mind and, hence, drives the acceptance of the organic food products. 

Although the limits of this research are related to the fact that it was conducted only in Montenegro, it should be noted that, according to the authors’ knowledge, it is the first research of this type conducted in the mentioned country. Additionally, the obtained results can be significant for research in other countries too, considering at least two reasons. First, the organic food market in Montenegro is still underdeveloped, which can stand for organic food markets in many countries. Secondly, most respondents in this survey have an average income and are price sensitive, while the segment of price-sensitive consumers exists in every country. A further limitation of this paper arises from the fact that there is not any difference between loyal and occasional buyers of organic food products nor between different consumer segments that may exist regarding their demographic or psychographic characteristics. In order to overcome these limitations, further studies on this topic should include respondents from several countries and analyze if there are differences in the influence of each of the four elements of the offer on the purchasing decision, observed by different consumer segments. Additionally, whether the relative influence of each of those four elements changes over time can be explored. Considering the fact that optimization of the offer is an indispensable prerequisite for organic food acceptance and organic market development, similar research should be conducted not only in developing but in developed countries as well.

## Figures and Tables

**Table 1:** An overview of the respondents’ characteristics.

Sample Characteristics	*N*	%	Sample Characteristics	*N*	%
**Gender**			**Purchase frequency**		
Male	417	39.7	Every day	180	11.0
Female	634	60.3	At least once a week	371	22.7
**Age structure**			1–3 times a month	317	19.4
15–25	394	37.5	Few times a year	183	11.2
26–40	378	36.0	Never	583	35.7
41–60	182	17.3			
More than 60	97	9.2			
**Level of education**			**Average monthly income**		
Primary school	68	6.5	Less than 500 euros	705	67.1
Secondary school	437	41.6	500–800 euros	242	23.0
Bachelor	470	44.7	800–1300 euros	86	8.2
Master’s Degree	59	5.6	1300–2000 euros	18	1.7
PhD	17	1.6	More than 2000 euros	0	0

**Table 2:** Reliability indexes’ values for the model specification.

Index	Value
Goodness of Fit Index (GFI)	0.951
Adjusted Goodness of Fit Index (AGFI)	0.9
Root Mean Square Error of Approximation (RMSEA)	0.08

**Table 3:** Estimation results of the structural equation model (SEM).

Exogenous Variable	Path Direction	Endogenous Variable	Estimate	Standard Error (SE)	Critical Ratio (CR)	Probability (P)
Attitudes	<---	Product	1.000			
Producer	<---	Product	0.932	0.046	20,212	***
Packaging	<---	Product	0.132	0.02	6,6	***
Purchase frequency	<---	Product	0.229	0.017	13.47	***
Importance of price height	<---	Price	0.329	0.046	7.191	***
Price/quality ratio	<---	Price	1.000	0.051	4.821	***
Distribution barriers	<---	Distribution	1.000			
Shop store	<---	Distribution	0.232	0.047	4.933	***
Modern media	<---	Promotion	1.000			
Traditional media	<---	Promotion	0.342	0.065	5.261	***
Promotion	<---	Purchasing decision	1.000			
Product	<---	Purchasing decision	0.137	0.029	4.724	***
Distribution	<---	Purchasing decision	0.194	0.034	5.705	***
Price	<---	Purchasing decision	1.000			

*** The regression coefficient, which is statistically significant with the level of significance of 1% (two-sided test).

**Table 4:** Estimated results of the structural equation model (SEM) for women and men.

Regression Coefficients			Female	Male
Attitudes	<---	Product	1.000	1.000
Producer	<---	Product	0.191	0.195
Packaging	<---	Product	0.173	0.237
Purchase frequency	<---	Product	−0.121	1.046
Importance of price height	<---	Price	−1.000	−1.000
Price/quality ratio	<---	Price	1.532	3.793
Distribution barriers	<---	Distribution	−1.379	−1.500
Shop store	<---	Distribution	0.203	0.576
Modern media	<---	Promotion	1.000	1.000
Traditional media	<---	Promotion	9.660	0.618
Promotion	<---	Purchasing decision	1.000	1.000
Product	<---	Purchasing decision	0.700	0.630
Distribution	<---	Purchasing decision	2.445	0.152
Price	<---	Purchasing decision	−4.620	−1.644

## Appendix A

**Table A1:** Previous research undergrounding the conceptual model of research.

References	Sample	Methodology	Object of Research
Ham et al. 2016 [33]	In person survey of 411 households in Croatia	Techniques of univariate and multivariate analysis	Relationship between organic food purchase and purchasing barriers (time, price, knowledge, attitudes)
McEachern & McClean 2002 [10]	Random sample of 200 respondents; conducted in Scotland	Correlation and principle component analysis	Attitudes towards organic food products; purchasing motivation;
Bezawada & Pauwels 2013 [44]	The store-level date retrieved from 75 outlets in the USA	The persistence modeling approach	How changes in promotion, price and product assortment affect organic and conventional food purchase
Aschemann-Witzel et al. 2013 [41]	A survey of 210 organic consumers in Germany	Techniques of univariate and multivariate analysis	How nutrition, health or risk reduction claims visible on product packaging affect organic food purchase
Iyer et al. 2016 [31]	Online survey of 249 respondents in the USA	Structural Equation Modeling (SEM)	The effect of economic-driven motives (price and value consciousness) and altruistic motives on green purchase intentions
Henryks & Pearson 2011 [51]	A snowball sample of 21 respondents in Australia	A grounded theory approach	Identifies the variables affecting consumer choice of retail outlet and finds the ones that play a significant role in organic food purchase
Islam, S. 2014 [50]	A survey of 646 random shoppers visiting retail shops in Canada	Multiple regression analysis	Examines the choice of retail outlet and consumers’ willingness to pay premium prices for organic food products
Hidalgo-Baz et al. 2017 [53]	Random sample consisting of 311 respondents in Spain	Linear regression model	Impact of different claims on organic food purchasing

## Appendix B—Questionnaire

This survey is designed in order to investigate the specificities of the optimization of the offer on the organic food market. Please, take a few minutes to complete it. The survey is anonymous, and the data will be used for the purpose of writing a research paper.

1. Gender:

(a) male
(b) female

2. Age:

(a) 15–25
(b) 26–40
(c) 41–60
(d) more than 60

3. Your level of education:

(a) primary school
(b) secondary school
(c) Bachelor
(d) Master’s Degree
(e) PhD

4. Your average monthly income:

(a) less than 500 €
(b) 500–800 €
(c) 800–1300 €
(d) 100–2000 €
(e) more than 2000 €

5. How often you buy organic food products:

(a) every day
(b) at least once a week
(c) 1–3 times a month
(d) few times a year
(e) never

If your answer to the question no. 5 is “never”, this is the end of a questionnaire for you. If you chose any other given answer, please proceed with questionnaire.

6. What is the type of organic food products that you buy most often:

(a) organic fruit and vegetables
(b) organic dairy products
(c) organic cereals
(d) organic meat products
(e) organic bread and pasta
(f) organic juices and other soft drinks
(g) organic baby food
(h) other _______________________(Please, specify)

7. In your opinion, what type of organic food products should be more available on the market (you can choose more answers):

(a) organic fruit and vegetables
(b) organic dairy products
(c) organic cereals
(d) organic meat products
(e) organic bread and pasta
(f) organic juices and other soft drinks
(g) organic baby food
(h) other _______________________(Please, specify)

8. Rate the following characteristics of the organic food products on a scale 1–5 (1—*completely disagree*, 2—*disagree*, 3—*indifferent*, 4—*agree*, 5—*completely agree*):

(a) They are healthier than conventional products	1 2 3 4 5
(b) They are suitable for the prevention and treatment of certain diseases	1 2 3 4 5
(c) They are free from GMO, pesticides, antibiotics and other additives	1 2 3 4 5
(d) They have higher nutritional value than conventional products	1 2 3 4 5
(e) They have better taste than conventional products	1 2 3 4 5
(f) Their production is not harmful for the environment	1 2 3 4 5
(g) Organic production assures animal welfare	1 2 3 4 5
(h) By purchasing of organic food products you can give support to the local producers	1 2 3 4 5

9. Which reason for purchasing organic food is most important for you:

(a) its positive impact on health
(b) its nutritional value
(c) taste
(d) habit
(e) to contribute to the environmental protection
(f) animal welfare
(g) I don’t know
(h) other __________________________(Please, specify)

10. Rate the following statements on a scale 1–5 (1—*completely disagree*, 2—*disagree*, 3—*indifferent*, 4—*agree*, 5—*completely agree*):

I do not buy organic food products in larger quantities because of:	
(a) high prices compared to my level of income	1 2 3 4 5
(b) high prices compared to the quality of these products	1 2 3 4 5
(c) they taste worse than conventional	1 2 3 4 5
(d) the available offer is scarce (not diversified)	1 2 3 4 5
(e) they are not sufficiently represented in the stores, so their purchase requires too much time	1 2 3 4 5
(f) I don’t know where to find them	1 2 3 4 5
(g) they have worse appearance than conventional products	1 2 3 4 5
(h) there is no real difference between organic and conventional products	1 2 3 4 5
(i) I don’t have enough information about characteristics of these products	1 2 3 4 5

11. From the items offered below, select the three most important that you consider when buying organic food products and rank them by importance from 1 to 3 (1—*the most important*, 2—*of less importance*, 3—*of the least importance*)

(a) price	1 2 3
(b) nutritional value	1 2 3
(c) impact on health	1 2 3
(d) taste	1 2 3
(e) diversity of the offer	1 2 3
(f) packaging appearance	1 2 3
(g) packaging size	1 2 3
(h) information about producer	1 2 3
(i) other _______________________________(Please, specify)	1 2 3

12. What would particularly encourage you to buy more organic food products:

(a) price reduction
(b) better quality of the products
(c) greater diversity of the offer
(d) better representation of these products in supermarkets and other food stores
(e) opening new stores specialized in the organic food selling
(f) better quality and diversity of its packaging
(g) in no case I would buy them in larger quantities than I do now

13. Where do you usually buy organic food products:

(a) in supermarkets
(b) in small shops
(c) in stores specialized for selling organic food
(d) at the markets
(e) directly from the producer
(f) on the organic food fairs
(g) over the Internet
(h) other ____________________________(Please, specify)

14. In which types of stores would you prefer organic products to be available for purchase on a permanent basis:

(a) in the supermarkets
(b) in the small stores
(c) wish there were more stores specialized in organic food selling
(d) in the markets
(e) internet shops
(f) other ____________________________(Please, specify)

15. Rate the following statements on a scale 1–5 (1—*not important at all*, 2—*not important*, 3—*indifferent*, 4—*important*, 5—*very important*)

It is very important for organic food purchasing if these products are:

(a) available in the shops near the place where I live	1 2 3 4 5
(b) available in the shops where I usually purchase food products	1 2 3 4 5
(c) available in the shops with long working hours	1 2 3 4 5
(d) available in the shops that have friendly personnel	1 2 3 4 5
(e) available in the shops that offer high value for money	1 2 3 4 5
(f) available in the shops with high quality purchase services	1 2 3 4 5

16. How much higher price of organic food products (compared to conventional ones) are you ready to pay:

(a) I am not ready to pay higher prices for organic food products
(b) 0%–20%
(c) 20%–40%
(d) 40%–60%
(e) 60%–80%
(f) 80%–100%

17. From which sources do you usually collect information about organic food products:

(a) Friends and family members
(b) TV shows
(c) TV and radio commercials
(d) social networks
(e) internet portals or specialized websites
(f) magazines
(g) flyers
(h) other ____________________(Please, specify)

18. In your opinion, which media are more convenient for informing consumers about specificities and benefits of organic production system:

(a) traditional media (such as TV, radio, newspapers, magazines, flyer etc.)
(b) modern media (Internet based media, such as social networks, emails, websites, pop-up advertisement, mobile marketing etc.)
(c) other ____________________(Please, specify)

19. In your opinion, which media are more convenient for informing consumers about available offer of the organic food products in the market:

(a) traditional media (such as TV, radio, newspapers, magazines, flyers etc.)
(b) modern media (Internet based media, such as social networks, emails, websites or pop-up advertisement, mobile marketing, etc.)
(c) other ____________________(Please, specify)

20. How do you determine if food products labeled as "organic" are truly organic?

(a) based on official certification mark placed on the packaging
(b) based on information about producer (I buy it from well-known organic producer)
(c) based on the trust in the shop
(d) based on the product appearance
(e) based on label "organic"
(f) other _______________________ (Please, specify)

21. Rate the following items on a scale 1 – 5 according to their significance in purchasing organic food products (1—*not important at all*, 2—*not important*, 3—*indifferent*, 4—*important*, 5—*very important*):

(a) the way organic food products are differentiated from conventional ones in the stores	1 2 3 4 5
(b) appearance and the size of packaging	1 2 3 4 5
(c) label design	1 2 3 4 5
(d) information displayed on the product packaging	1 2 3 4 5

22. Rate how much important are the following items to you when choosing the organic producer whose products you will buy (1—*not important at all*, 2—*not important*, 3—*indifferent*, 4—*important*, 5—*very important*)

(a) the producer possesses the appropriate product quality certificate	1 2 3 4 5
(b) the producer has years of experience in organic food production	1 2 3 4 5
(c) the producer packs its products in recognizable packaging with a specific label	1 2 3 4 5
(d) the producer has a variety of organic food products in its offer	1 2 3 4 5
